# Draft Genome Sequence of a New Zealand Rangipo Strain of *Mycobacterium tuberculosis*

**DOI:** 10.1128/genomeA.00657-16

**Published:** 2016-07-07

**Authors:** Micheál Mac Aogáin, Sanjay S. Gautam, James E. Bower, Indira Basu, Ronan F. O’Toole

**Affiliations:** a Department of Clinical Microbiology, School of Medicine, Trinity College Dublin, St. James’s Hospital, Dublin, Ireland; b Breathe Well Centre, School of Medicine, University of Tasmania, Hobart, Australia; c LabPlus, Auckland City Hospital, Auckland, New Zealand

## Abstract

The Rangipo genotype of the *Mycobacterium tuberculosis* complex has been associated with a number of tuberculosis (TB) outbreaks in New Zealand. We report here the draft whole-genome sequence of a representative isolate of this strain.

## GENOME ANNOUNCEMENT

Tuberculosis (TB) is a leading cause of infectious mortality worldwide, killing approximately 1.5 million people each year (1). In an earlier work, we determined that New Zealand isolates of the *Mycobacterium tuberculosis* complex were dominated by lineage 4 (Euro-American: 37.8%; 95% confidence interval [CI], 33.6 to 42.2%), followed by lineage 1 (Indo-Oceanic: 22.6%; 95% CI, 19.1 to 26.5%), lineage 2 (East Asian: 19.5%; 95% CI, 16.2 to 23.3%), and lineage 3 (East-African Indian: 17.7%; 95% CI, 14.5 to 21.3%) (2). In lineage 4 (Euro-American), 75 of the 184 isolates were in clusters (40.8%; 95% CI, 33.9 to 48.0%). The largest of these clusters belonged to the so-called Rangipo genotype based on 24-locus mycobacterial interspersed repetitive-unit–variable-number tandem-repeat (MIRU-VNTR) typing.

In 2001, an analysis of an outbreak of TB in New Zealand, which occurred between November 1996 and May 2000, was reported (3). Forty-three of the 61 TB cases were confirmed to belong to the outbreak by IS*6110*-based restriction fragment length polymorphism (RFLP) typing of the isolates, with the remaining 18 cases determined by epidemiological contact tracing. One of the patients had served a prison sentence in the Tongariro/Rangipo Prison in 1998 and, as a result, the strain subsequently became referred to as the Rangipo strain (4).

Here, the genomic DNA of a Rangipo isolate, NZ494, was sequenced using an Illumina MiSeq instrument. A total of 2,128,439 paired-end reads were mapped to the *M. tuberculosis* strain H37Rv reference genome (accession no. AL123456/NC_000962) by the Burrows-Wheeler Aligner (5). This yielded an average read depth of 45-fold, covering 99.5% of the H37Rv genome. Variants relative to the H37Rv reference genome were called using the SAMtools analysis suite, and variant annotation was performed using SnpEff (6, 7). A 4,292,219-bp draft genome assembly of 179 contigs was assembled *de novo* using the SPAdes assembler (version 3.7) (8).

A total of 851 variant sites were identified relative to the H37Rv genome and consisted of 782 single-nucleotide variants (SNVs) and 69 insertions/deletions. Five hundred eighty-seven of the variants were nonsynonymous, of which 549 were SNVs and 38 were insertions/deletions. The genome of Rangipo isolate NZ494 did not display high-confidence single-nucleotide polymorphisms in genes correlating with antimicrobial drug resistance when analyzed using the PhyResSE database (9), which was consistent with the isolate’s drug-susceptible phenotype.

An outbreak of TB in 2002 in Hawke’s Bay involving 19 active cases of Rangipo TB was associated with a high rate of transmission, as determined by the presentation of TB disease or latent TB infection in 16.4% and 20.0%, respectively, of household or other close contacts (6). The reasons underlying the potentially high infectivity of the Rangipo strain are not known. Therefore, investigations on the virulence and antigenic determinants of the Rangipo genotype of *M. tuberculosis* are needed to generate a better understanding of its inherent propensity to transmit and cause disease in relation to other strains of the bacterium.

### Nucleotide sequence accession number.

This whole-genome shotgun project has been deposited at DDBJ/EMBL/GenBank under the accession no. LXWG00000000.

## References

[1] World Health Organization 2015 Global tuberculosis report. World Health Organization, Geneva, Switzerland http://apps.who.int/iris/bitstream/10665/191102/1/9789241565059_eng.pdf.

[2] Yen S, Bower JE, Freeman JT, Basu I, O’Toole RF 2013 Phylogenetic lineages of tuberculosis isolates in New Zealand and their association with patient demographics. Int J Tuberc Lung Dis 17:892–897. doi:10.5588/ijtld.12.0795. 23635796

[3] De Zoysa R, Shoemack R, Vaughan R, Vaughan A 2001 A prolonged outbreak of tuberculosis in the North Island. N Z Public Health Rep 8:1–8.

[4] McElnay C, Thornley C, Armstrong R 2004 A community and workplace outbreak of tuberculosis in Hawke’s Bay in 2002. N Z Med J 117:U1019. 15475989

[5] Li H, Durbin R 2010 Fast and accurate long-read alignment with Burrows-Wheeler transform. Bioinformatics 26:589–595. doi:10.1093/bioinformatics/btp698. 20080505 PMC2828108

[6] Cingolani P, Platts A, Wang LL, Coon M, Nguyen T, Wang L, Land SJ, Lu X, Ruden DM 2012 A program for annotating and predicting the effects of single nucleotide polymorphisms, SnpEff: SNPs in the genome of *Drosophila melanogaster* strain w1118; iso-2; iso-3. Fly (Austin) 6:80–92. doi:10.4161/fly.19695. 22728672 PMC3679285

[7] Li H, Handsaker B, Wysoker A, Fennell T, Ruan J, Homer N, Marth G, Abecasis G, Durbin R, Genome Project Data Processing S 2009 The Sequence Alignment/Map format and SAMtools. Bioinformatics 25:2078–2079. 19505943 10.1093/bioinformatics/btp352 PMC2723002

[8] Bankevich A, Nurk S, Antipov D, Gurevich AA, Dvorkin M, Kulikov AS, Lesin VM, Nikolenko SI, Pham S, Prjibelski AD, Pyshkin AV, Sirotkin AV, Vyahhi N, Tesler G, Alekseyev MA, Pevzner PA 2012 SPAdes: a new genome assembly algorithm and its applications to single-cell sequencing. J Comput Biol 19:455–477. doi:10.1089/cmb.2012.0021. 22506599 PMC3342519

[9] Feuerriegel S, Schleusener V, Beckert P, Kohl TA, Miotto P, Cirillo DM, Cabibbe AM, Niemann S, Fellenberg K 2015 PhyResSE: a Web tool delineating *Mycobacterium tuberculosis* antibiotic resistance and lineage from whole-genome sequencing data. J Clin Microbiol 53:1908–1914. doi:10.1128/JCM.00025-15. 25854485 PMC4432036

